# Engineering dosimetric excellence in total body irradiation: Tomotherapy‐driven protocols for precision and reproducibility

**DOI:** 10.1002/pro6.70049

**Published:** 2026-01-19

**Authors:** Sandeep Singh, Dipesh, Supratik Sen, Abhay Kumar Singh, Mahipal, Manindra Bhushan, Jaskaran Singh Sethi, David K. Simson, Munish Gairola

**Affiliations:** ^1^ Department of Radiation Oncology, Division of Medical Physics Rajiv Gandhi Cancer Institute and Research Center New Delhi India; ^2^ Department of Physics GLA University Mathura Uttar Pradesh India; ^3^ Department of Radiation Oncology Dr. B. Borooah Cancer Institute Guwahati Assam India; ^4^ Department of Radiation Oncology Rajiv Gandhi Cancer Institute and Research Center New Delhi India

**Keywords:** dose homogeneity, gamma analysis, helical delivery, hematopoietic stem cell transplantation, IGRT, in‐vivo dosimetry, patient‐specific QA, quality assurance, total body irradiation

## Abstract

**Problem:**

The clinical feasibility, dosimetric reproducibility, and in vivo accuracy of a tomotherapy‐based total‐body irradiation (TBI) protocol in a large cohort of patients undergoing hematopoietic stem cell transplantation (HSCT) is evaluated.

**Methods:**

Patients (128 patients with hematological malignancies undergoing TBI with helical tomotherapy) were simulated in dual orientations (head‐first and feet‐first supine) to accommodate extended anatomical lengths. Plans were generated as separate upper and lower components with structured gradient overlap zones and then composited for delivery. Quality assurance included an ArcCHECK 3D diode array for patient‐specific gamma analysis (3%/3 mm criteria), point‐dose verification, and in vivo dosimetry. The gamma pass rates, regional dose deviations, and Pearson correlation coefficients were analyzed. Statistical evaluation was performed using the Shapiro–Wilk test, Levene's test, and ANOVA.

**Results:**

All treatment plans met the institutional constraints for planning target volume (PTV) coverage and organs at risk sparing. The mean D95% of the PTV consistently exceeded 95% of the prescribed dose, ensuring robust target coverage. This reflects excellent conformity with the planned dose distribution. Region‐wise gamma pass rates exceeded 97% in all areas except junctions, where rates were slightly lower owing to dose gradients. The point‐dose agreement showed tight clustering within the ±3% range, with the pelvis exhibiting the most significant positive shift. The in vivo optically stimulated luminescence dosimeter dose remained within the clinically acceptable range of 0.8–1.2 Gy. A strong interregional correlation (*r* > 0.90) was observed, confirming reproducibility. ANOVA identified statistically significant but clinically acceptable regional dose variations (*p* = 0.030).

**Conclusion:**

Tomotherapy‐based TBI demonstrated high dosimetric precision, reproducibility, and workflow efficiency. This protocol offers a clinically reliable approach for modern TBI delivery in transplant conditioning.

## Graphical abstract

• Objective: Assess helical Tomotherapy‐based TBI for clinical feasibility, dosimetric reproducibility, and in‐vivo accuracy using dual‐orientation simulation and patient‐specific QA.

• Methodology: Implementation of ArcCHECK 3D diode array, ionization chamber verification, and in‐vivo dosimetry with OSLDs at 16 anatomical sites to ensure precise dose delivery.

• Results & Conclusion: Achieved >97% gamma pass rates and ±3% point‐dose agreement, demonstrating high dosimetric precision and reproducibility, validating the protocol for clinical use in HSCT conditioning.

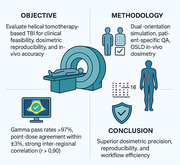

## 1 1.INTRODUCTION

Total body irradiation (TBI) remains the cornerstone of preparative regimens for hematopoietic stem cell transplantation (HSCT), particularly for hematologic malignancies such as acute lymphoblastic leukemia (ALL), acute myeloid leukemia (AML), chronic myeloid leukemia (CML), and various forms of lymphoma.^1^, ^2^, ^3^ The primary goal of TBI is to achieve uniform myeloablation and immunosuppression to facilitate donor engraftment and reduce the risk of malignant relapse.^4^, ^5^ However, the technical challenges involved in delivering a homogeneous dose across the entire body while minimizing exposure to organs at risk (OAR) make TBI one of the most complex procedures in clinical radiotherapy. Historically, TBI has been administered using large, manually configured, extended SSD (source to surface distance) techniques or fixed beam arrangements on C‐arm linear accelerators, often resulting in suboptimal dose uniformity and significant inter‐field junction uncertainties.^4^, ^5^ These limitations are similar to those encountered in craniospinal irradiation (CSI), where field‐matching errors can result in substantial overdosage or underdosage at junctions between adjacent treatment fields.^6^, ^7^, ^8^, ^9^ In recent decades, CSI planning has evolved significantly, transitioning from conventional simulator‐based techniques to modern approaches, such as 3D‐chemotherapy and radiation therapy, intensity‐modulated radiotherapy (IMRT), and volumetric modulated arc therapy (VMAT), allowing for more precise dose sculpting and reduction of long‐term sequelae in pediatric patients with medulloblastoma or primitive neuro‐ectodermal tumors. Similarly, TBI techniques have been advanced by leveraging the capabilities of newer technologies such as helical tomotherapy to address the intrinsic dosimetric challenges of full‐body irradiation.

Tomotherapy combines the principles of IMRT with a helical delivery mechanism and image‐guided radiotherapy (IGRT), making it uniquely suited for treatments that require extensive longitudinal coverage and high conformality.^10^, ^11^, ^12^ Unlike traditional VMAT or C‐arm‐based techniques, tomotherapy delivers radiation in a slice‐by‐slice manner with continuous couch translation, thereby eliminating the need for field junctions and associated dose inhomogeneities.^13^ This allows for a highly conformal dose to the entire body while simultaneously sparing critical structures such as the lungs, kidneys, and lenses, which is an essential consideration in pediatric and adolescent populations at risk of late radiation‐induced toxicities.^14^, ^15^

In 2021, following the installation and commissioning of a helical tomotherapy system at our institution, we will use this platform for TBI in patients undergoing HSCT for hematological malignancies. Over a period of four years (2021–2025), we treated a cohort of 128 patients using this technique. This transition enhanced our planning precision and workflow efficiency and enabled us to better adhere to dose constraints for critical organs, particularly in pediatric and adolescent cases. This study reports our institutional experience with tomotherapy‐based TBI in this large patient cohort, focusing on dosimetric outcomes, clinical feasibility, and the evolution of planning strategies in comparison with earlier CSI and conventional TBI approaches.

## 2 MATERIALAND METHODS

### 2.1 Tomotherapy

The Radixact™ Tomotherapy system (Accuray Inc., Sunnyvale, CA) is a ring‐gantry‐based, helical delivery platform equipped with a fixed, flattening filter‐free (FFF) 6 MV linear accelerator mounted on a continuously rotating gantry, housed within an 85 cm bore, and capable of delivering a nominal dose rate of up to 1,000 cGy/min at isocenter. It delivers radiation slice by slice as the patient's couch translates through the bore, allowing for fully helical IMRT. The system employs a binary multi‐leaf collimator (MLC) consisting of 64 pneumatically driven leaves, each 6.25 mm wide at the isocenter, providing highly modulated beam shaping across a longitudinal field length of up to 40 cm. The maximum field width in the craniocaudal direction can be set to 1.0, 2.5, or 5.0 cm, with the latter allowing faster treatment times at the expense of slightly reduced modulation. The actual field width used in this study was 5 × 40 cm^2^ with dynamic jaw features. Beam delivery was synchronized with continuous gantry rotation and couch movement, enabling precise dose painting across large treatment volumes without requiring field junctions. Image guidance was performed using an integrated kV fan‐beam CT (FBCT) system mounted orthogonally to the treatment beam, allowing pretreatment volumetric imaging for patient setup verification and adaptive planning. The system was calibrated to deliver 1 cGy/MU at Dmax for a source‐to‐axis distance of 85 cm.^16^, ^17^

### 2.2 Patient Selection Criteria

The 128 patients included in this study underwent TBI between September 2021 and March 2025, following the clinical commissioning of the helical Tomotherapy Radixact™ system at our institute. All patients were diagnosed with hematological malignancies, including ALL,^18^ AML,^19^ CML,^20^ or various subtypes of lymphoma,^21^ and were scheduled for TBI as part of their conditioning regimen for hematopoietic stem cell transplantation. All medically fit patients eligible for bone marrow transplantation (BMT), irrespective of age were included in the study. These included individuals with a history of prior radiation therapy, such as prophylactic cranial irradiation for ALL or radiation for other malignancies, as well as those who had previously undergone failed BMT. However, patients with severe organ dysfunction, such as interstitial lung disease, advanced pulmonary fibrosis, end‐stage renal disease, or a history of radiotherapy, were excluded from the study cohort. The treatment was delivered exclusively using the Radix Act tomotherapy platform. In each case, comprehensive dosimetric parameters were extracted and analyzed, including target volume coverage, dose homogeneity across longitudinal body segments, hotspot volumes, and OAR doses to the lungs and kidneys.^22^, ^23^ The patient parameters are listed in Table 1.

**TABLE 1:** Disease and other parameters for patients.

Parameters	<Lower Threshold |Mean±Standard Deviation| Upper Threshold>
**Age (Years)**	< 4 | 26.01 ± 13.76 | 50 >
**Sex**	
Male	89
Female	39
Patient Height (cm)	< 96 | 133.3 ± 25.5 | 180 >
**Diagnosis**	
ALL (including B‐ALL subtype)	73
AML	35
B‐Cell Lymphoma	1
CLL	1
CML	10
Fanconi Anemia	1
Myelodysplastic Syndrome	3
Pancytopenia	1
T‐ALL	3
**Prescription dose**	
Average (Gy)	< 2 | 4.43 ± 4.24 | 12 > (Median value = 4.0 Gy)
Without Brain Boost	2 Gy in a single fraction or 4 Gy in 2 fractions, or 8 Gy in 8 fractions
With Brain Boost	8 Gy in 8 fractions to the whole body, 12 Gy to the entire brain

Abbreviations: AML, acute myeloid leukemia; CML, chronic myeloid leukemia; ALL, lymphoblastic leukemia.

### 2.3 Simulation and Contouring

CT simulations were performed for all 128 patients in both head‐first supine (HFS) and feet‐first supine (FFS) orientations to achieve full‐body coverage with a 30 cm overlap at the junction. Scans were acquired using a Somatom Go.Sim CT Scanner (SIEMENS Healthineers, Germany) with a 5 mm slice thickness, offering high‐resolution imaging and soft tissue clarity. Owing to the tomotherapy system's 130 cm treatment limit, dual‐position scans were required. Both scans had an overlap length of 20 cm, resulting in an additional imaging dose of 3 mSv (Millisievert). This value was derived from scanner‐reported dose‐length product (DLP) values multiplied by region‐specific conversion factors (k‐values).^24^ However, 3 mSv represents a modest diagnostic exposure, which is negligible relative to the therapeutic dose range of 8–12 Gy delivered during TBI, and therefore does not meaningfully contribute to radiation risk. Nevertheless, to minimize any additional exposure, low‐dose CT acquisition protocols were employed, and repeat scans were strictly avoided. The patients were immobilized with thermoplastic masks for brain treatment and vacuum cushions for body support, with fiducials and a lead wire at the HFS–FFS junction to aid registration. The scanner's 1,600 mm range and rapid rotation enabled complete, artifact‐free acquisition, as verified by a dual‐technologist checklist. Anesthesia was reserved for uncooperative pediatric or claustrophobic patients, whereas most children tolerated the simulation without it. The growth plates were not contoured, parents were counselled, and informed consent was obtained. An eleven‐point safety checklist preceded each simulation, covering identity verification, height measurement (activating the long‐length protocol if >120 cm), and confirmation of the immobilisation devices.^25^ The patients were positioned reproducibly, three fiducials were placed for isocenter verification, and the laser alignment was confirmed before marking the skin references. The CT protocols were site‐ and length‐specific, with the field of view, slice thickness, exposure, and contrast tailored as required. Each checklist step was signed off, and scanning was initiated only when all criteria were met.

The DICOM datasets were imported into Accuray Precision treatment planning system (TPS) v3.3.1.3 (Accuray Inc., Sunnyvale, CA, USA), where HFS and FFS scans were reviewed for orientation, completeness, and absence of artifacts. Key anatomical landmarks, particularly in overlapping regions, were verified to support accurate registration and contouring. Rigid registration was performed using fiducial and anatomical markers, and manual alignment was refined using automated tools. Final alignment was confirmed using multi‐planar views and 3D reconstructions, ensuring junction accuracy within ±2 mm. The planning target volume (PTV) was generated with a 3 mm inner margin from the body contour.^26^

This inner margin was selected to accommodate minor setup uncertainties under daily image‐guided radiotherapy (IGRT), supported by prior findings that contracting 3–5 mm from the body contour is an accepted practice for improving the dosimetric accuracy while minimizing the skin dose buildup.^14^, ^27^ Furthermore, studies have shown that without daily imaging, the required PTV margins can exceed these values (up to 6.4 mm in craniocaudal directions),^28^ confirming that our chosen 3 mm margin remains appropriate, given our IGRT workflow. This approach ensures robust coverage of subcutaneous tissues while mitigating artificial dose inflation at the skin surface.

In our protocol, the lungs, kidneys, liver, brain, lenses, and gonads were routinely contoured as the primary OAR. High doses to these OARs were avoided. The mean lung doses were limited to 7 Gy and the kidneys to 8 Gy, and also ensuring no hotspots exceeded 110%.^29^, ^30^ The maximum lens dose was restricted to less than 10 Gy. The testes were typically excluded from junctional strips, and under unavoidable circumstances, their maximum dose was restricted to < 3 Gy in the 2 Gy regimen. The cranial and testicular sanctuary regions, particularly those relevant to ALL, were not spared. Both adult patients and the parents of pediatric patients were explicitly informed of the potential short‐ and long‐term risks, including cataracts and infertility, and appropriate consent was obtained.

### 2.4 Treatment Planning

All treatment plans used 6 MV FFF photon beams at a dose rate of 1,000 MU/min. Two separate plans were created for each patient, PLAN_UPPER (HFS) and PLAN_LOWER (FFS), which covered the entire body length. Dose calculations were performed using the highest available grid resolution (1.17 mm × 3 mm × 1.17 mm) in the TPS. This resolution minimizes the uncertainties in steep dose‐gradient regions such as junctions and ensures an accurate representation of the dose distribution. The calculation time remains clinically acceptable at this resolution. The modulation factor used ranged from 2.5 to 3.0, depending on the patient's anatomy and plan complexity. This range balances plan quality and delivery efficiency, an MF below approximately 1.8–2.0 may compromise dose uniformity, while values above 3.0 prolong beam‐on time without meaningful dosimetric benefit.^31^, ^32^ In the context of TBI with helical tomotherapy, other groups have identified an optimal modulation factor (MF) around 2.4 when combined with a pitch of 0.287, offering strong homogeneity and acceptable beam‐on durations.^33^ Accordingly, our patient‐specific MF selection reflects this evidence and ensures robust target coverage and plan conformity while maintaining clinically acceptable treatment times.

**FIGURE 1:**
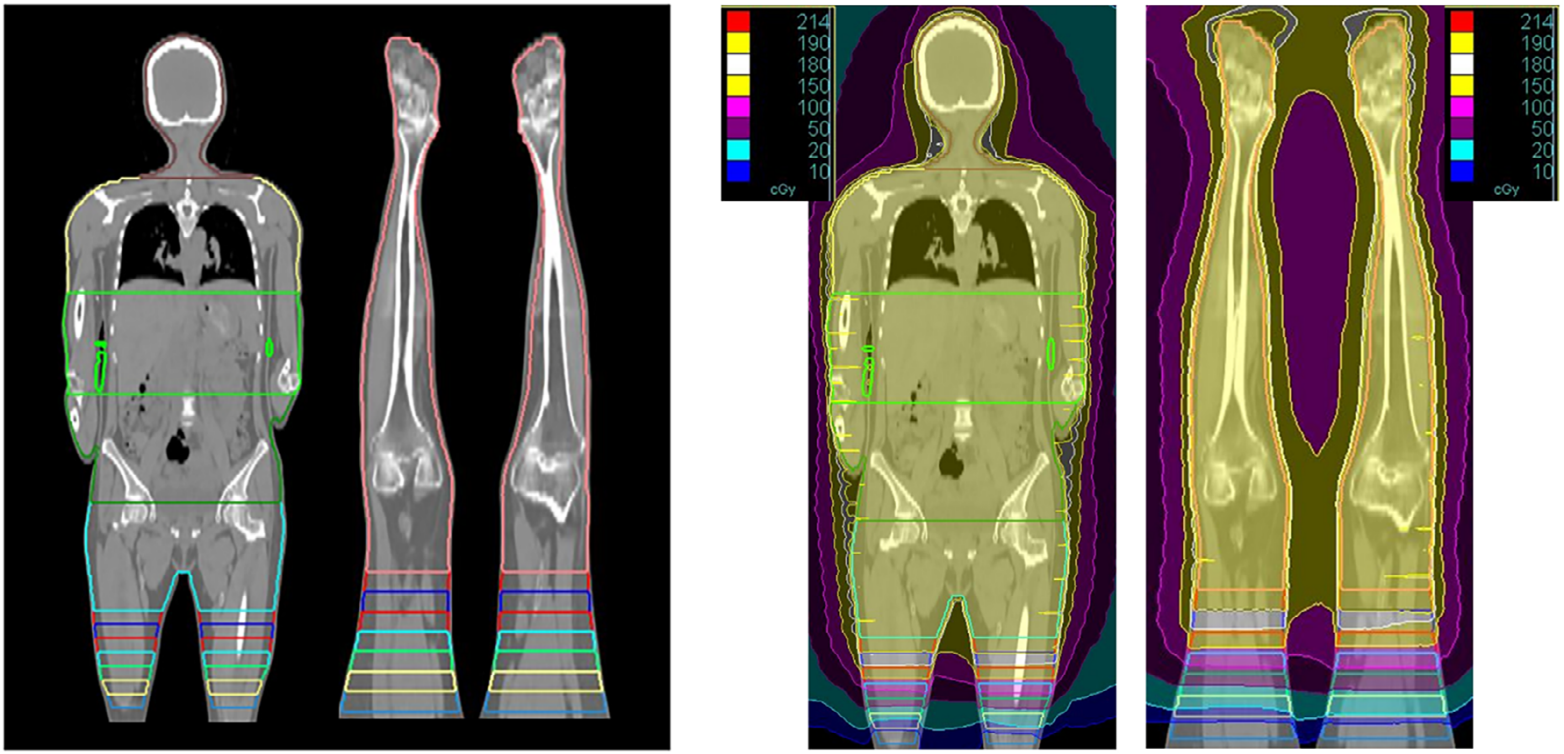
Subdivision of the whole PTV into small regions and overlapping junction (Left), dose wash for both the plans (Right), PTV, planning target volume.

The PTV was subdivided into the following anatomical segments: brain, thorax, abdomen and overlap for HFS, and lower limbs and overlap for FFS. A seven‐layer dose gradient (95% to 5%) (Figure 1 (Left)) was applied across the overlap region, contoured in descending order on HFS and ascending on FFS to ensure smooth junction blending. Each plan was independently optimized and summed to form the final composite plan (Figure 1 (Right)).

### 2.5 Dosimetry

**FIGURE 2:**
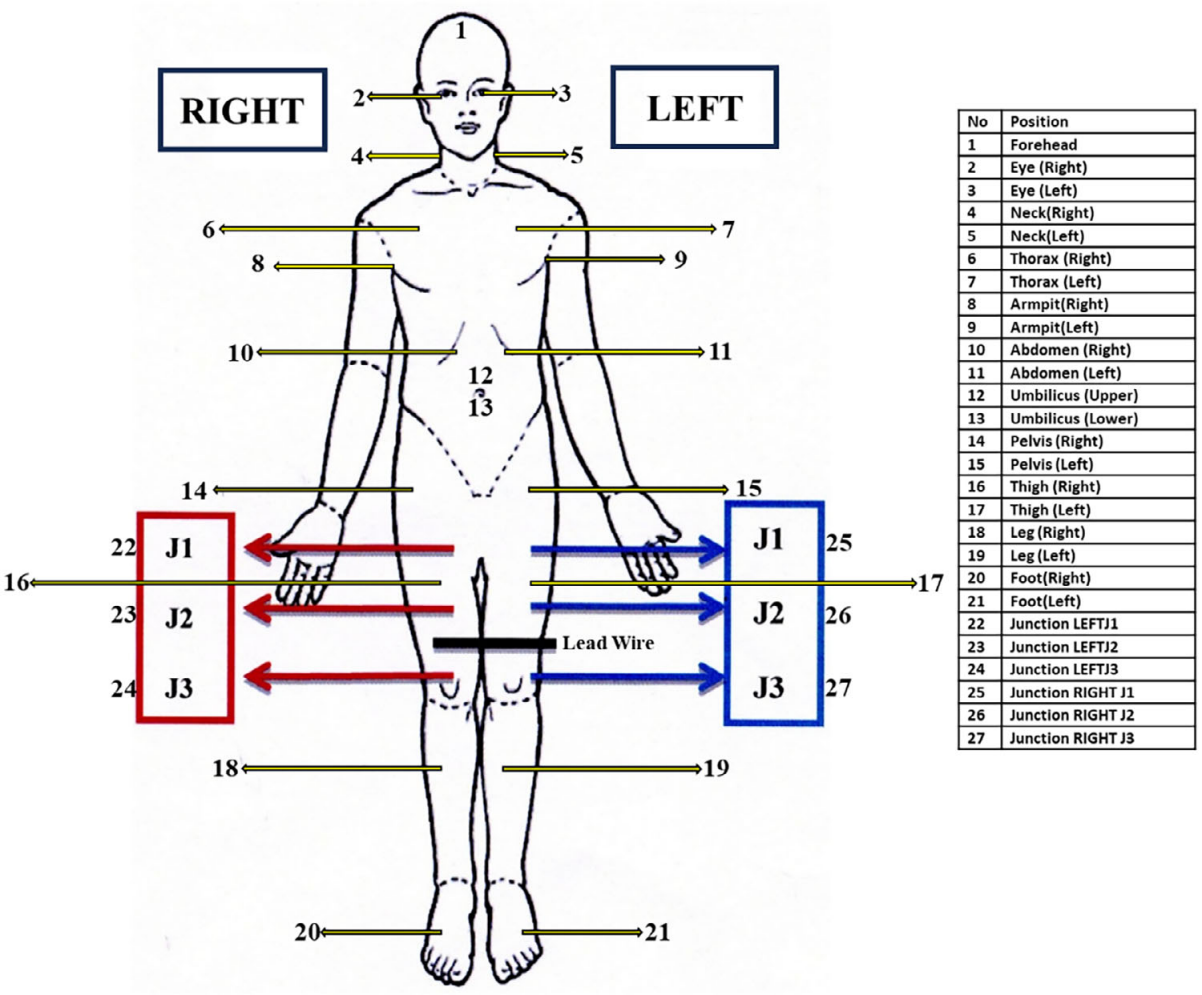
Position of anatomical sites for OSLD placement for in vivo dosimetry, OSLD, optically stimulated luminescence dosimeter.

Before treatment delivery, patient‐specific quality assurance (QA) was performed using the ArcCHECK 3D diode array system (Sun Nuclear Corporation, Melbourne, FL), applying 3%/3 mm gamma index criteria in accordance with TG‐218 recommendations for each region separately.^34^ We also verified the point dose using an ionization‐based chamber for all areas, except the junction region (owing to the presence of dose heterogeneity). Additionally, in vivo dosimetry was conducted using optically stimulated luminescence dosimeters (OSLDs) with a myOSLchip (RadPro International GmbH, Germany) placed at multiple anatomical landmarks during the first treatment fraction to verify the actual dose delivery(Figure 2).^35^

Each OSLD was individually calibrated in a 6 MV photon beam at a 100 cm SSD using a solid‐water phantom, following the protocol recommended by AAPM TG‐191.^36^ A reference dose of 200 cGy was measured using a calibrated Farmer‐type ionization chamber traceable to the IAEA secondary standard, and calibration coefficients were derived for each detector relative to the chamber measurement. The energy and angular dependences of the OSLDs were evaluated in solid‐water phantoms by varying the depth (5–20 cm) and gantry angle (0°–180°). The measured variation was within ±3% for energy dependence and ±2% for angular response, consistent with TG‐191 data and manufacturer specifications. The combined measurement uncertainty, incorporating calibration, reproducibility, and reader variability, was estimated as ±5% (k = 2). For validation in heterogeneous geometries, commissioning measurements were also performed using an anthropomorphic Alderson phantom, where the OSLD readings at the cranial, thoracic, and pelvic sites were compared with the reference ionization chamber data. Agreement was within ±3%, confirming the reliability of OSLDs for in vivo dosimetry in TBI.

These integrated quality assurance procedures validated the accuracy, reproducibility, and clinical robustness of the tomotherapy‐based TBI protocol across the patient cohorts.

### 2.6 Data Processing and Statistical Analysis

**FIGURE 3:**
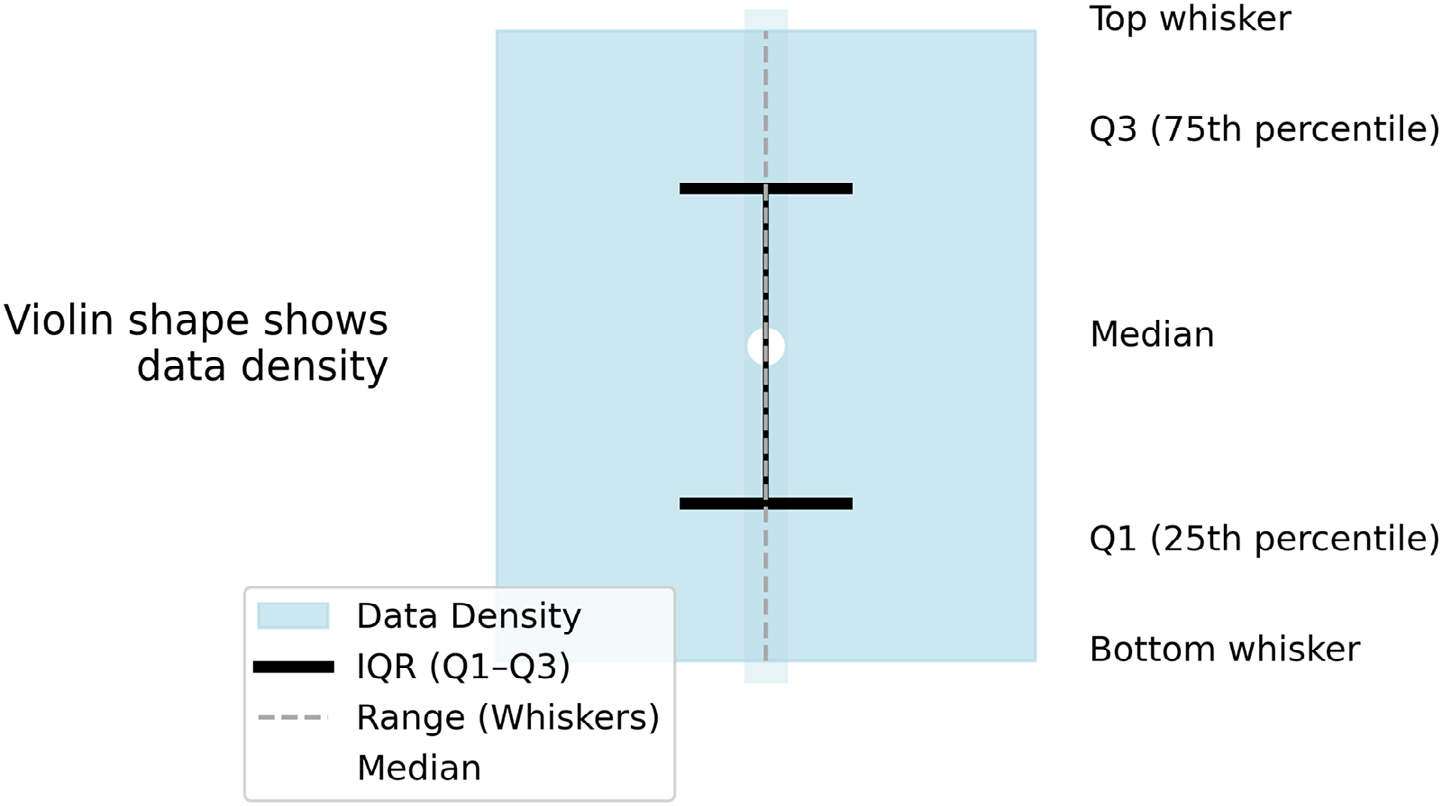
Schematic of a violin plot. The shaded area represents the density of data across the range of values. The bold black bars indicate the interquartile range (IQR: 25th to 75th percentile), with the central white dot marking the median. Thin dashed lines (whiskers) extend to the minimum and maximum values within 1.5× IQR.

Descriptive statistics (mean, standard deviation, minimum, maximum, and interquartile ranges) were computed for each region (Figure 3: legend to read violin plot). The normality of the data distributions was evaluated using the Shapiro–Wilk test, while the assumption of equal variance across groups was assessed using Levene's test. A one‐way analysis of variance (ANOVA) was performed to assess whether statistically significant differences existed in the mean values among the five anatomical regions. All analyses were conducted using Python version 3.10.9.^37^ Data analysis and visualization were performed using Pandas, Matplotlib, SciPy, and Seaborn libraries.

## 3 RESULTS

The plan evaluation confirmed robust PTV coverage and adherence to institutional dose constraints. Across all anatomical subregions, mean V95% was ≥95%, ranging from 95.2% ± 1.6% in the junction to 97.2% ± 1.1% in the brain. Similarly, D95% values were consistently >95% (95.1%–96.8%), and D98% remained >93% across regions. Hotspot indices were well‐controlled, with Dmax between 107.8% and 112.5% of prescriptions and V107% consistently <3% (1.2%–2.4%). The OAR analysis confirmed compliance with institutional constraints. The mean lung dose was 6.4 ± 0.5 Gy, and the mean kidney dose was 7.2 ± 0.6 Gy, both remaining comfortably below their respective tolerance thresholds of 7 Gy and 8 Gy, as summarized in Table 2. These results demonstrate that the tomotherapy‐based TBI protocol achieved comprehensive target coverage while maintaining OAR sparing within a clinically acceptable range.

**TABLE 2:** Dosimetric results across anatomical subregions (mean ± SD) and OAR doses. All regions met institutional criteria.

Region / OAR	V95% (%)	D98% (%)	D95% (%)	Dmax (% of Prescription)	V107% (%)	Mean OAR Dose (Gy)
Brain	97.2 ± 1.1	94.8 ± 0.9	96.8 ± 0.7	108.2 ± 2.1	1.4 ± 0.6	–
Thorax	96.4 ± 1.3	94.2 ± 1.0	96.0 ± 0.8	109.5 ± 2.5	1.7 ± 0.7	Lungs: 6.4 ± 0.5
Abdomen	96.0 ± 1.5	94.0 ± 1.2	95.8 ± 0.9	110.0 ± 2.4	1.8 ± 0.8	Kidneys: 7.2 ± 0.6
Pelvis	95.7 ± 1.4	93.8 ± 1.1	95.5 ± 0.9	111.0 ± 2.6	2.0 ± 0.9	–
Legs	96.9 ± 1.2	94.6 ± 1.0	96.6 ± 0.7	107.8 ± 2.0	1.2 ± 0.5	–
Junction	95.2 ± 1.6	93.6 ± 1.2	95.1 ± 1.0	112.5 ± 2.7	2.4 ± 0.9	–

Abbreviation: OAR, organ at risk.

A quantitative evaluation of the absolute dose deviations in the overlapping regions was conducted in accordance with the institutional plan acceptability criteria. When normalized to a prescription dose of 1 Gy (100%), the absolute cold spot was quantified as 0.942 ± 0.011 Gy (range:    0.928~0.958Gy), representing the D98% range across junctions. The D_max_ values were found to be 1.087 ± 0.012 Gy (range:1.076~1.120 Gy), all within the upper limit of 1.20 Gy (120%). Additionally, the V107%, reflecting the volume receiving more than 107% of the prescription dose, corresponded to an absolute range of 0.018~0.035 Gy ( 0.021 ± 0.0105 Gy). These results confirm the adherence to institutional constraints and affirm the dosimetric integrity of field matching across all anatomical subregions.

**FIGURE 4:**
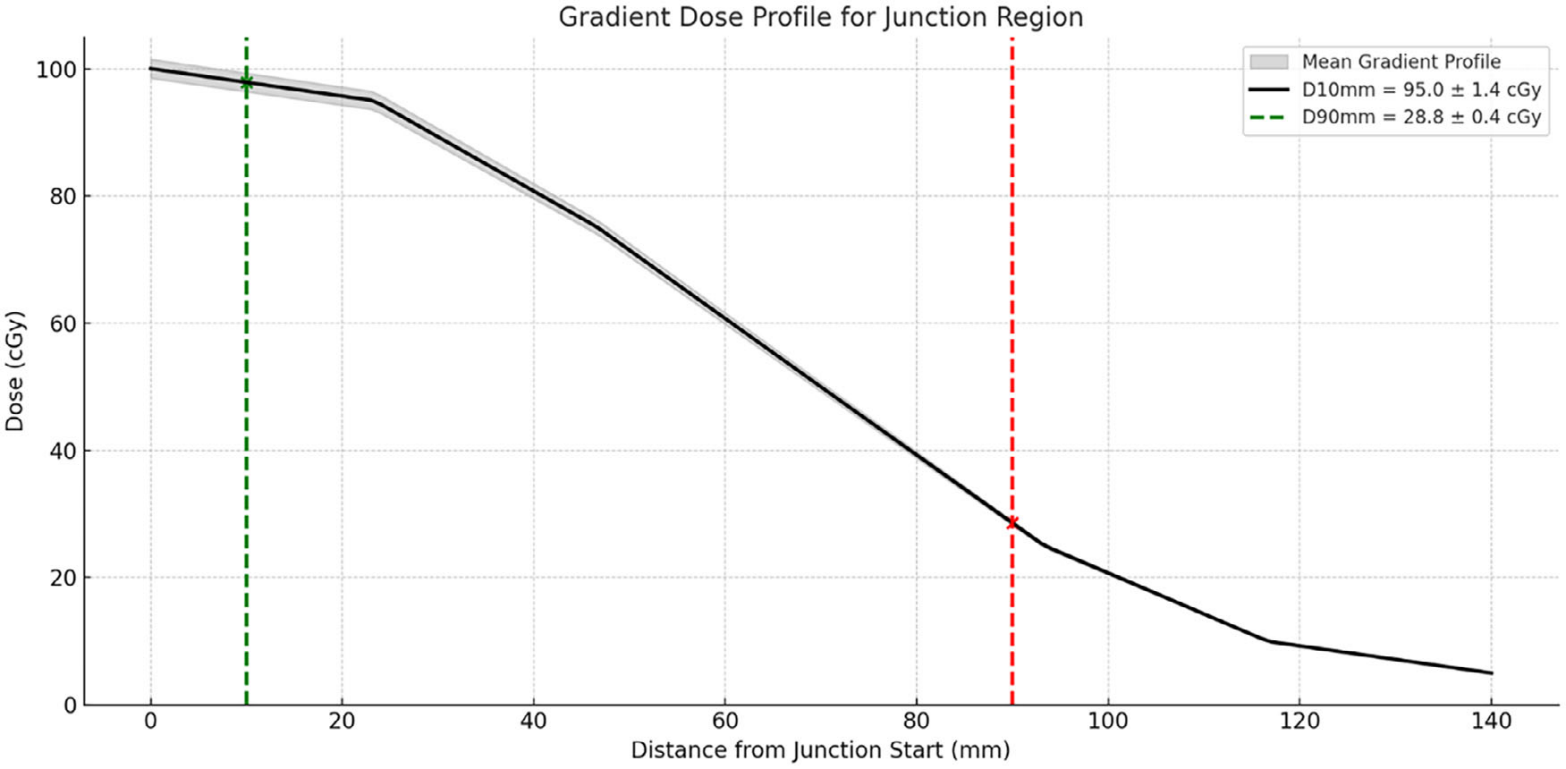
Gradient dose profile for the junction region spanning 14 cm, divided into seven 2 cm layers. The black line represents the mean dose across 128 patients, with the shaded area indicating ±1.5% standard deviation (this was chosen instead of ±1 SD to enhance visual interpretability, because significantly low variability (<2%) at the junction would render a ±1 SD band indistinguishable from the mean curve. The ±1.5 SD envelope, therefore, provided a clearer depiction of inter‐patient variability while maintaining clinical accuracy). Vertical dashed lines mark the D10mm (green) and D90mm (red) positions, corresponding to 10 and 90 mm from the start of the junction, respectively. Mean dose values at these points were 95.0 ± 1.4 and 28.8 ± 0.4 cGy, respectively. The profile demonstrates a controlled and reproducible gradient fall‐off, supporting robust junction optimization in TBI.

To quantitatively evaluate the robustness of the seven‐layer gradient junction design, we analyzed the dose distribution across the junction span (14 cm) in 128 patient plans. The gradient region was structured with seven 2 cm layers, modulating the dose from 95% to 5% of the prescription. Key robustness metrics were assessed at 10 mm (D10mm) and 90 mm (D90 mm) from the start of the junction. The mean dose at D10mm was 95.0 ± 1.4 cGy and 28.8 ± 0.4 cGy at D90mm, indicating a controlled and reproducible dose fall‐off across the junction interface. These values confirmed the spatial accuracy of the dose modulation, ensuring a smooth transition between adjacent treatment fields and reducing the risk of both overdoses and underdoses in the junction region. The gradient profiles are shown in Figure 4, supporting the consistency and robustness of the junction strategy for clinical TBI delivery.

Sensitivity analysis was also performed to evaluate the effects of setup errors in the junction region. Therefore, a CT scan of the RANDO phantom was acquired similarly to that described above for patient simulation. Two treatment plans were generated, one using the HFS CT dataset and the other using the FFS dataset. To assess delivery uncertainties within the overlap region, systematic couch shifts of ±5 mm in the longitudinal (y) direction were simulated in the plan using the TPS. These simulated displacements enabled the evaluation of positional deviations in the dose distribution and the robustness of each planning strategy against setup errors. Shifts are expressed as (±a, ±b), where ±a corresponds to couch movement for the HFS plan and ±b corresponds to movement for the FFS plan. Their effects on the regions of overdosage are quantified in Table 3.

**TABLE 3:** Sensitivity analysis of setup errors in the junction region using the RANDO phantom. Simulated longitudinal couch shifts (±a, ±b) were introduced in the TPS, where a represents displacement in the HFS plan and b denotes displacement in the FFS plan. The values represent the relative maximum dose (%) observed in the overlap region compared to the reference (0,0) shift.

	b		
a	−0.5	0.0	0.5
**−0.5**	105.5	103.2	104.8
**0.0**	102.8	100.0	101.9
**0.5**	104.6	103.7	103.9

**FIGURE 5:**
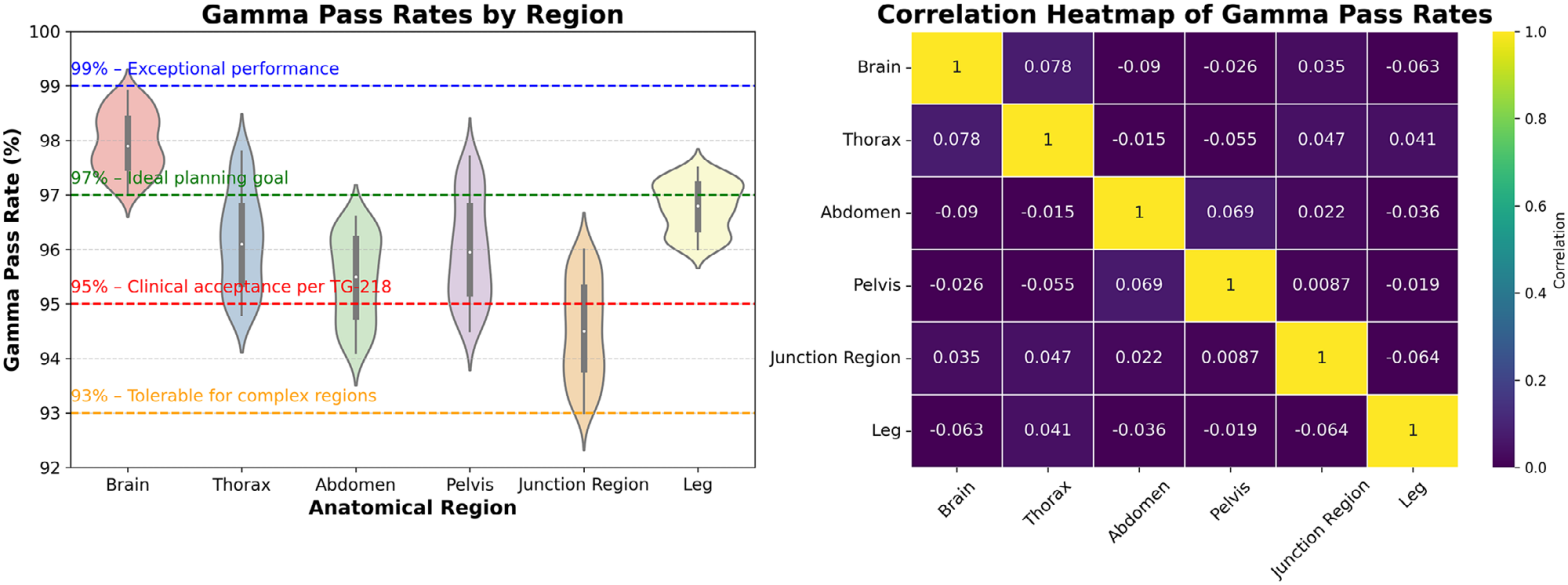
Distribution of gamma pass rates (%) across different anatomical regions, with threshold lines for clinical acceptance criteria (91%–99%) (Left). Heatmap illustrating Pearson correlation coefficients between gamma pass rates of anatomical regions (Right).

Before treatment, all patients underwent independent fluence verification to ensure precise radiation delivery. Gamma pass rates were region‐specific, with values recorded as follows: Brain region, 98.0% ± 0.5% (range: 97.0%~98.9%); Thorax region, 98.0% ± 0.9% (range:94.8%~97.8>%); Abdomen, 95.4% ± 0.8% (range:94.1%~96.6%); Pelvis, 96.0% ± 0.9% (range: 94.5%~97.7%); Junction region, 94.5% ± 0.9% (range: 93.0%~96.0%); and Leg region, 96.8% ± 0.5% (range:96.0%~97.5%). The junction region exhibited lower gamma pass rates than other areas, primarily owing to the steep dose gradients and inherent dose heterogeneity encountered at the anatomical transition points, as shown in Figure 5.

After applying a false discovery rate (FDR) correction to control for multiple comparisons, several interregional associations remained statistically significant. The strongest correlations were observed between the adjacent anatomical regions, including the brain and thorax (*r* = 0.874), thorax and abdomen (*r* = 0.862), and abdomen and pelvis (*r* = 0.841). Moreover, distal but symmetric regions, such as the pelvis‐leg (*r* = 0.802), also retained significance. More distant region pairs (e.g., brain–leg, brain–pelvis) demonstrated weaker associations and did not remain significant after FDR adjustment. These findings confirm that strong, spatially contiguous correlations were preserved even under multiple comparison controls, highlighting the reproducibility of the delivered dose patterns across anatomically related regions.

**FIGURE 6:**
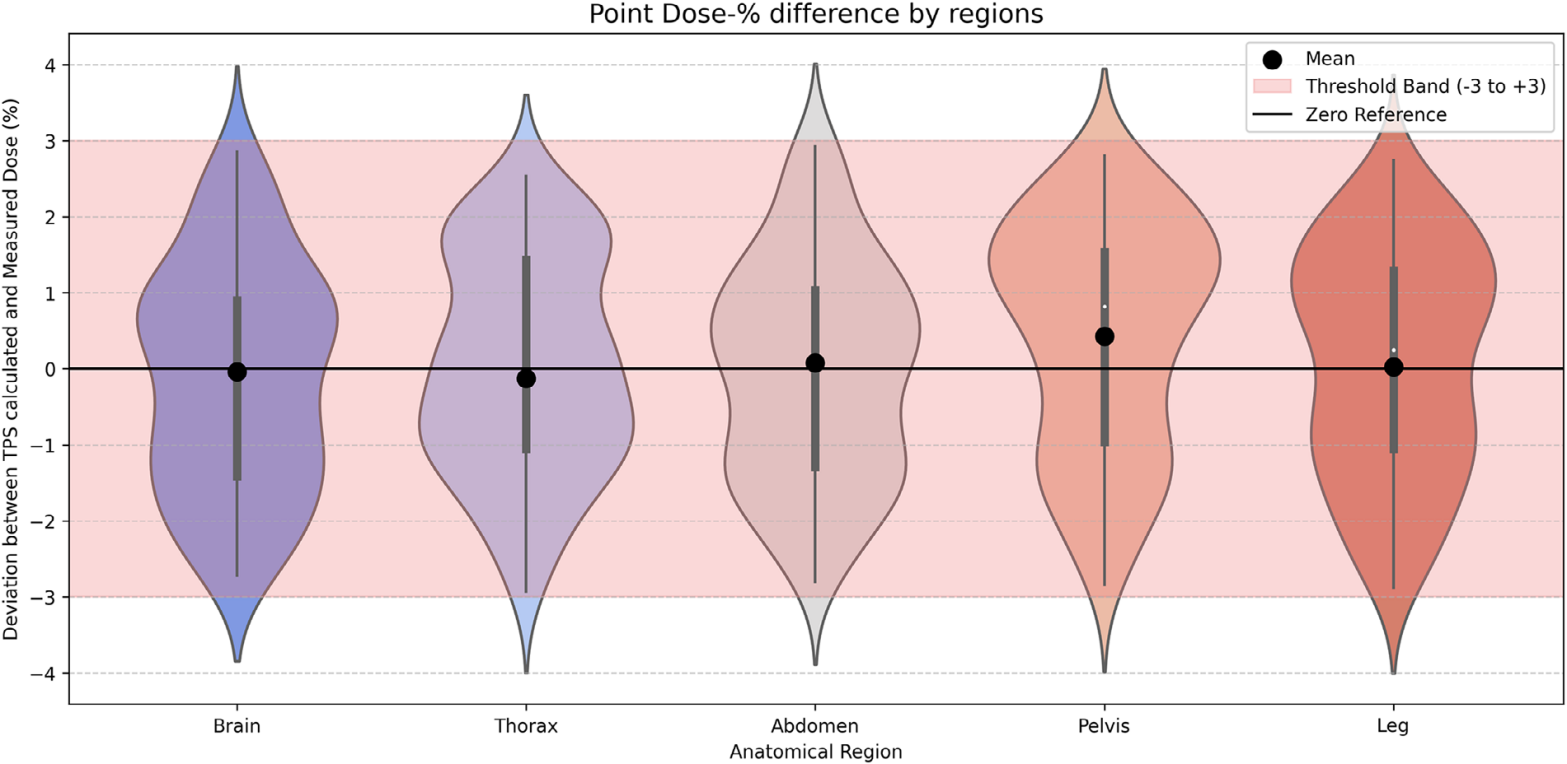
Schematic of the distribution of percentage differences between TPS calculated doses and measured point doses across anatomical regions. Black dots represent mean values for each region. The red shaded area indicates the clinically acceptable deviation range (±3%).^38^, ^39^ The black horizontal line at 0 denotes perfect agreement between calculated and measured doses.

For point‐dose measurements in the brain region, the observed values were −0.12% ± 1.49 % (range: ‐1.41%~0.90%), with a moderate degree of variability around zero and a non‐normal distribution (Shapiro–Wilk test, *p* < 0.01). In the thorax region, values showed 0.03% ± 1.41% (range: ‐1.05%~1.43%), reflecting the lowest variability among all areas, although the distribution was slightly skewed and violated normality (*p* < 0.01). Measurements from the abdomen region were characterized as −0.04% ± 1.44% (range:‐1.30%~1.04%), with symmetric variability around zero and a mild deviation from normality (*p* = 0.017). The pelvis region exhibited a pronounced rightward shift in values, summarized as 0.43% ± 1.51% (range:‐0.97%~1.54%), significantly contributing to the overall ANOVA result. Finally, the leg region showed values of 0.08% ± 1.49% (range: ‐1.06%~1.30%), with variability similar to other anatomical areas, and the distribution again significantly departed from normality (*p* < 0.01) (Figure 6). To further characterize the practical significance of this finding, effect size metrics were calculated. The eta‐squared (η^2^) was 0.0024, consistent with a significantly small effect, suggesting that only 0.24% of the total variance in dose differences could be attributed to anatomical region. The epsilon‐squared (ε^2^) was negative (–0.0038), which is interpreted as effectively zero and indicative of a negligible effect. Collectively, these results demonstrate that the TPS dose calculation accuracy remained consistent and robust across different anatomical sites, with no region showing a disproportionate deviation from the measured values.

Nonparametric analyses were also conducted on the percentage differences between the TPS‐calculated and measured point doses across anatomical regions. Mann–Whitney *U* tests revealed no statistically significant differences between any regional pair (*p* > 0.05), indicating consistent dose agreement. The Kruskal–Wallis test confirmed the absence of global variation (*H* = 2.84, *p* = 0.584). Although the Friedman test approached significance *(χ* ^2^ = 9.44, *p* = 0.051), it did not reach the statistical threshold, suggesting a possible trend toward regional variability. These results collectively indicate uniform TPS measurement agreement across the brain, thorax, abdomen, pelvis, and legs.

ANOVA confirmed a statistically significant difference in the mean values across the regions (Table 4). These findings emphasize the necessity for region‐specific QA thresholds and reinforce the clinical relevance of anatomical stratification in treatment assessment.

**TABLE 4:** Descriptive statistics of normalized dose differences across anatomical regions.

Region	Mean	Median	IQR	Std Dev	Min	Q1 (25%)	Median (Q2)	Q3 (75%)	Max
Brain	−0.12	0.04	2.44	1.49	−2.72	−1.41	−0.01	0.90	2.85
Thorax	0.03	0.09	2.26	1.41	−2.93	−1.05	−0.06	1.43	2.53
Abdomen	−0.04	0.86	2.41	1.44	−2.80	−1.30	0.10	1.04	2.92
Pelvis	0.43	1.12	1.79	1.51	−2.84	−0.97	0.82	1.54	2.80
Leg	0.08	0.27	2.21	1.49	−2.88	−1.06	0.25	1.30	2.74

**FIGURE 7:**
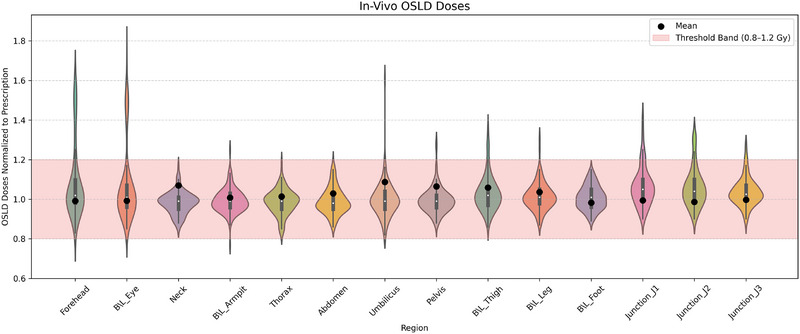
Illustration of the distribution and density of OSLD dose measurements at 16 anatomical sites, normalized to the prescribed dose (Normalized Dose = OSLD measured dose/Prescription Dose). The shaded pink area represents the clinically acceptable threshold band (0.8–1.2 Gy), indicating the target dose range. Black dots mark the mean dose for each region. Regions such as the forehead, bilateral eye, and pelvis show greater variability, while others, such as the umbilicus, BL thigh, and junctions J1–J3, display tighter distributions within the threshold.

The distribution of OSLD‐measured doses across anatomical sites is depicted in Figure 7, revealing both central tendencies and variability within each region. Sites such as the forehead, bilateral thighs, and junctions J1–J3 demonstrated narrow distributions, indicating consistent and reproducible dose delivery. Contrarily, regions such as the abdomen and thorax showed broader dose spreads, suggesting higher inter‐patient variability, which may be influenced by anatomical complexity or positioning differences.

When controlling for patient height, partial correlation analysis confirmed that interregional associations remained robust after Bonferroni adjustment. The strongest correlations were observed between anatomically adjacent or symmetric sites, such as junction segments (J1–J2, *r* = 0.782; J2–J3, *r* = 0.642) and bilateral sites (forehead‐eye, *r* = 0.896; thigh‐leg, *r* = 0.371), all of which remained statistically significant (*p* < 0.05). Distant anatomical pairs showed weaker or nonsignificant associations after correction for multiple tests. These findings indicate that the high reproducibility of OSLD measurements was not solely attributable to anthropometric scaling by patient height but instead reflected consistent dose delivery patterns across regions. Importantly, the correlations have now been interpreted as associations suggesting reproducibility rather than as independent variables, aligning with the reviewer's caution. (Refer to additional data provided in the Supplementary File).

In our cohort, the engraftment rate was 95%. At a median follow‐up of 6 months, 74% of patients were in complete remission, and 90% were alive. Early toxicities included mucositis in 46% of patients—predominantly Grade 2—and diarrhea in 20%. No parotitis or pneumonitis was observed. These clinical outcomes provide important context for the dosimetric advantages of our tomotherapy‐based TBI protocol.

**FIGURE 8:**
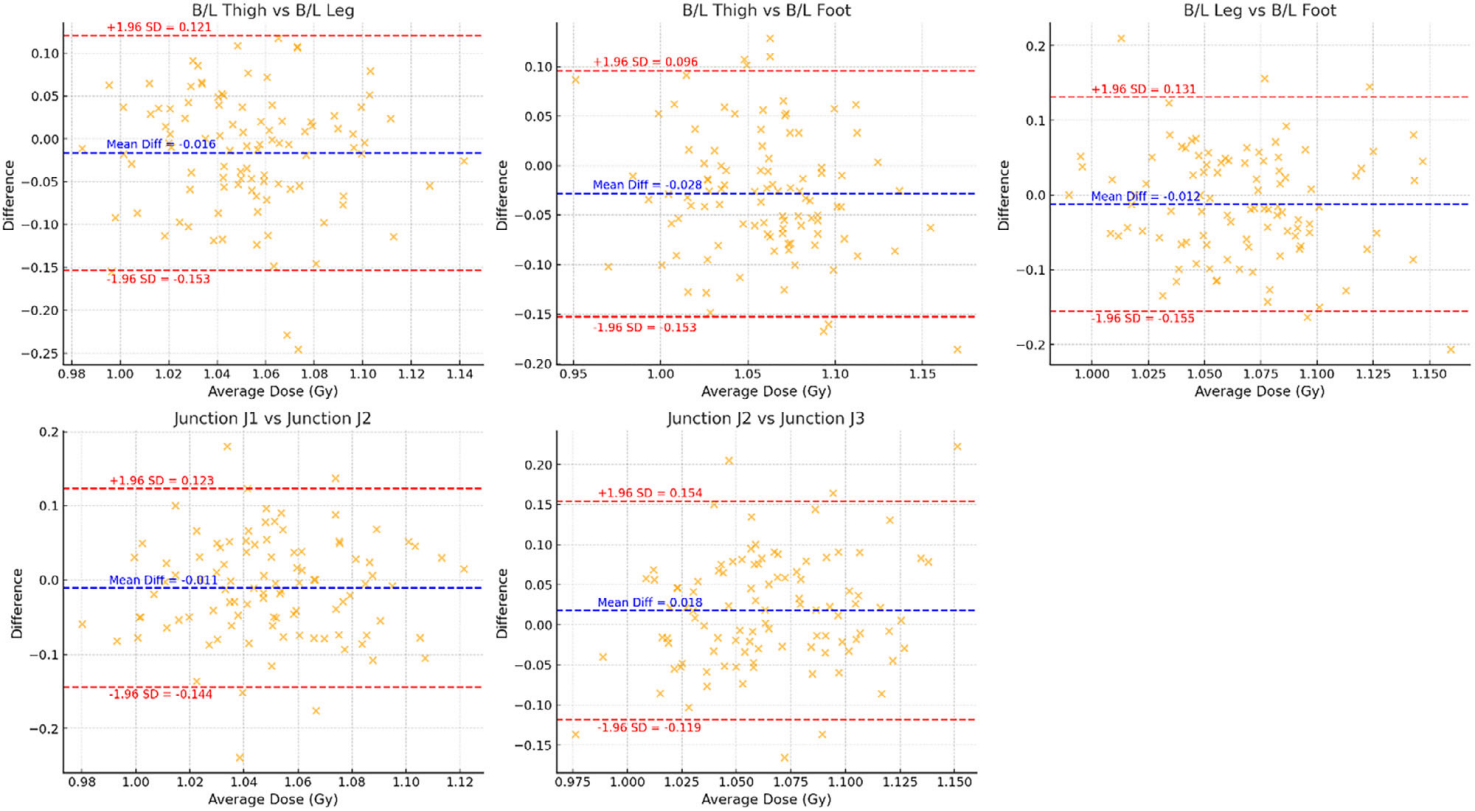
Bland–Altman Plot comparing dose agreement between anatomically paired regions. Each subplot illustrates the agreement between OSLD‐measured doses from two symmetric and adjacent anatomical sites. The central dashed blue line represents the mean difference (bias), whereas the red dashed lines indicate the 95% limits of agreement (±1.96 standard deviations). The plots reveal minimal bias and narrow agreement limits for junction regions (e.g., J1–J2), suggesting high dosimetric reproducibility. Comparisons involving distal limb sites (e.g., thigh–leg, thigh–foot) show wider variability, warranting attention to positioning or setup consistency.

To further assess the agreement of OSLD‐measured doses between anatomically symmetric and adjacent regions, Bland–Altman analysis was performed for five representative region pairs. As illustrated in Figure 8, the mean differences for all the pairs were close to zero, indicating a negligible systematic bias. Junction regions such as J1–J2 and J2–J3 demonstrated the narrowest limits of agreement, reinforcing the high reproducibility and spatial coherence of dose delivery across the overlapping field junctions. However, region pairs involving the distal limbs (e.g., B/L Thigh vs. B/L Foot) exhibited broader variability, suggesting a higher sensitivity to positioning or anatomical heterogeneity. These findings complement the high Pearson correlation coefficients observed previously and provide a more robust characterization of both relative association and absolute agreement, supporting the overall dosimetric consistency of the TBI protocol while highlighting areas where quality assurance measures may be optimized.

## 4 DISCUSSION

We evaluated the feasibility and clinical robustness of helical tomotherapy for TBI in 128 patients who underwent hematopoietic stem cell transplantation for hematologic malignancies. This protocol integrates dual‐orientation simulation, patient‐specific fluence verification, and in vivo dosimetry using optically stimulated luminescent dosimeters to establish a comprehensive QA framework for reproducible dose delivery.^40^, ^41^

**FIGURE 9:**
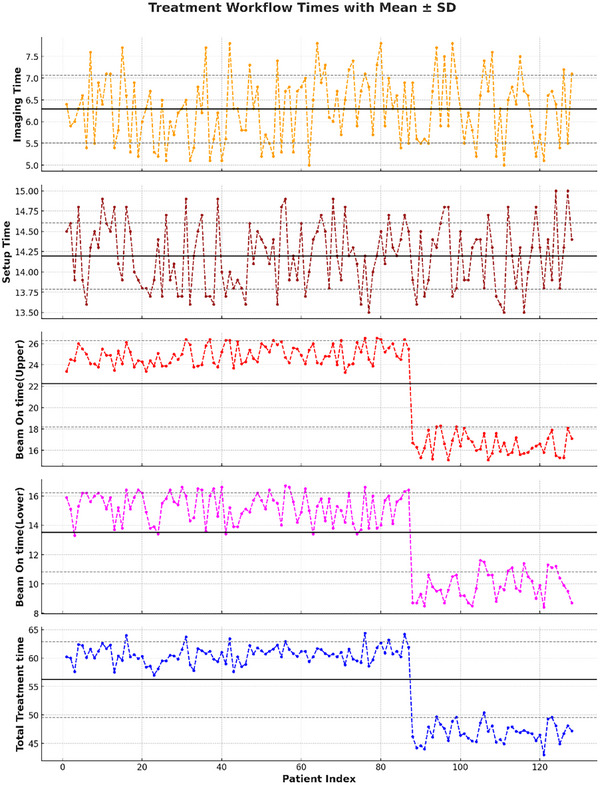
Temporal profile of radiotherapy workflow components across 128 patients. Each subplot depicts a key time metric: Imaging Time (orange), Setup Time (brown), Beam‐on Time for Upper (red) and Lower (magenta) body segments, and total treatment time (blue). Solid black lines represent the cohort mean, with dashed gray lines showing ±1 standard deviation, thereby reflecting inter‐patient variability. A distinct reduction in beam‐on and total treatment times is evident when shifting from 2 Gy per fraction to 1 Gy per fraction, whereas imaging and setup times remained stable across patients, indicating procedural uniformity irrespective of dose per fraction.

Compared with conventional C‐arm LINAC‐based TBI, tomotherapy offers a distinct advantage in terms of overall treatment efficiency by minimizing setup complexity. Traditional LINAC‐based TBI typically involves multiple isocenters owing to the limited field size, which requires frequent patient repositioning and manual field matching. These steps significantly increase the treatment time and introduce cumulative setup uncertainties, particularly at junction zones. Helical tomotherapy overcomes these challenges by eliminating field junctions through continuous couch translation and synchronized gantry rotation, enabling a smooth longitudinal dose distribution.^42^ Although the actual beam‐on time in tomotherapy may be slightly longer per fraction owing to beam modulation, the elimination of multiple setup stages and reliance on automated IGRT reduces the total session time and enhances workflow efficiency. This streamlined delivery is particularly beneficial in high‐volume clinical settings, enabling reproducible treatment with minimal interruptions and a reduced risk of setup‐related delays. Our analysis demonstrated highly consistent imaging and setup durations across all patients, with mean values of 6.3 min (range: 5.0–7.8 min) and 14.2 min (range: 13.5–15.0 min), respectively (Figure 9). Conversely, the beam‐on and total treatment times showed clear divergence according to the fractionation regimen. Patients prescribed 2 Gy per fraction required longer delivery, with mean beam‐on times of 24.9 min for the upper body and 15.4 min for the lower body, yielding an average total session duration of 60.3 min (range: 57.0–64.4 min). By comparison, patients treated with 1 Gy per fraction demonstrated reduced beam‐on times (16.8 min upper, 9.6 min lower) and a shorter overall session time averaging 46.8 min (range: 43.0–50.4 min). Statistical analysis confirmed these differences: mean beam‐on time was significantly higher in the 2 Gy cohort (40.3 ± 3.5 min) compared with the 1 Gy cohort (26.4 ± 2.8 min, *p* < 0.001). Similarly, total treatment duration was significantly prolonged in the 2 Gy group (60.3 ± 4.1 min) relative to the 1 Gy group (46.8 ± 3.6 min, *p* < 0.001). However, imaging and setup times did not differ significantly between the regimens (*p* > 0.05). Collectively, these results demonstrate that fraction size directly influences delivery efficiency, with the 1 Gy protocol providing shorter sessions and thereby greater operational flexibility.

We performed a retrospective comparison using data from our institution's pre‐2021 LINAC‐based extended SSD TBI program (34 patients). The mean beam‐on time with LINAC was (42.5 ± 5.2) min per side (85 ± 8) min in total, and total session duration (including imaging, setup, and patient handling) averaged (95 ± 10) min. In comparison, tomotherapy achieved substantially shorter delivery, with mean total session times of (60.3 ± 4.1) min in the 2 Gy cohort, representing a 37% reduction (Table 5). This efficiency translates into an estimated staff‐time saving of approximately 35 min per patient, with potential gains in throughput and a reduced setup‐related burden for both patients and staff. These findings are consistent with published reports, where extended SSD LINAC TBI requires 80–100 min per session, whereas tomotherapy TBI is typically completed within 35–60 min.

**TABLE 5:** Mean ± standard deviation (SD) of radiotherapy workflow components across 128 patients. Imaging and setup times were highly consistent with low variability, while beam‐on times and total treatment times showed greater inter‐patient variation.

Parameter	Mean (Minute)	SD (Minute)	Mean ± SD
Imaging Time	6.29	0.78	6.29 ± 0.78
Setup Time	14.20	0.41	14.2 ± 0.41
Beam On time(Upper)	22.24	4.05	22.24 ± 4.05
Beam On time(Lower)	13.51	2.70	13.51 ± 2.7
Total Treatment time	56.23	6.64	56.22 ± 6.64

All treatment plans met institutional criteria, with a mean gamma pass rate of 98%. Although slightly reduced gamma pass rates were observed in the junction regions, the spatial dose correlations remained high across all anatomical sites (r > 0.90), indicating strong reproducibility. In‐vivo OSLD verification showed all mean doses within the 0.8–1.2 Gy clinical threshold band and <2% standard deviation. Point‐dose analysis identified region‐specific shifts, particularly in the pelvis and legs, which were attributed to anatomical and beam‐path variability. Although these differences were statistically significant (ANOVA, *p* = 0.030), they remained clinically acceptable.^43^, ^44^, ^45^

To explore potential differences between pediatric and adult patients, the cohort was stratified into two groups: pediatric (<18 years) and adult (≥18 years). The analysis showed comparable dosimetric outcomes between the two groups. In pediatric patients, the mean lung dose was (6.3 ± 0.4) Gy and the mean kidney dose was (7.1 ± 0.5) Gy, values that were closely aligned with those in adults (6.5 ± 0.5) Gy and (7.3 ± 0.6) Gy, respectively. Both subgroups met the institutional tolerance thresholds. The PTV coverage indices also demonstrated no significant differences between age groups, confirming that protocol consistency was maintained across body sizes. While these results indicate that the tomotherapy‐based TBI approach provides uniform dosimetric quality for both children and adults, we still acknowledge that long‐term clinical follow‐up is essential in pediatric patients to monitor late effects such as growth impairment and endocrine dysfunction, as highlighted in prior studies.

The combined use of high‐resolution simulation, image‐guided adaptive planning, and multi‐tiered QA ensures that the full TBI pipeline, from simulation to beam delivery, is technically sound and clinically reproducible. This is particularly critical in pediatric and adolescent populations, where the long‐term sequelae of radiation are of greater concern, and precision is paramount for preserving organ function and quality of life. This single‐institution experience demonstrates that helical tomotherapy offers reliable, conformal TBI delivery with minimal interpatient variability. The protocol's integration of simulation, image guidance, and multitiered QA supports its broader adoption, particularly in pediatric and high‐risk transplant populations, where dose precision is critical for long‐term outcomes.

Our dosimetric results aligned with emerging data from other institutions. For instance, Konishi et al. (Japan) confirmed the feasibility of IMRT‐TBI using tomotherapy in a clinical setting,^46^, ^47^ while a European study comparing helical and static junction planning showed comparable target coverage and robustness.^48^ Moreover, a multicenter dosimetric analysis reported consistent OAR dose reductions with helical tomotherapy across multiple centers.^49^ Taken together, these studies suggest that our findings are confined to local protocols or equipment and reflect broader trends in optimized tomotherapy‐based TBI planning.

This study demonstrated the strong dosimetric reproducibility and technical robustness of the tomotherapy‐based TBI protocol. However, several limitations should be acknowledged to inform future research. The most important limitation was the absence of detailed long‐term clinical outcome data. Although early indicators, such as engraftment success, remission status, and acute toxicity, are favorable, comprehensive longitudinal follow‐up is required to evaluate relapse rates, late toxicities, and overall survival. Consequently, it remains uncertain whether the observed dosimetric advantages ultimately translate into durable clinical benefits. This analysis was designed primarily for dosimetric validation. Although the preliminary clinical outcomes were encouraging, these results were not sufficient to establish correlations among OAR doses, target coverage, and clinical endpoints. Larger cohorts with extended follow‐up are necessary to rigorously assess the influence of dosimetry on toxicity profiles, engraftment dynamics, and disease control.

Additionally, the study was inherently limited by its single‐institution design, which may introduce selection bias and constrain the generalizability of the findings. Institutional protocols, patient characteristics, and technical expertise can vary significantly. Therefore, to reinforce external validity, we propose the development of prospective, multi‐institutional trials, perhaps through collaborative groups such as ESTRO or ILROG, using harmonized dosimetric benchmarks and patient‐reported outcomes. Such studies would be instrumental in validating the robustness of the protocol, establishing normative OAR dose profiles, and confirming the reproducibility in diverse clinical settings.

Furthermore, the intrafractional motion during extended tomotherapy delivery was not monitored. Given the prolonged beam‐on times, particularly in 2 Gy per fraction regimens, small patient movements could result in deviations from the planned dose distribution, potentially affecting both target coverage and OAR sparing. Incorporating motion management tools such as surface‐guided radiation therapy or real‐time imaging can provide added assurance of treatment fidelity. These limitations underscore the importance of ongoing refinement of TBI protocols. Future studies should prioritize the integration of long‐term clinical follow‐up, inter‐institutional collaboration, and intrafractional motion assessment to comprehensively evaluate the clinical impact of tomotherapy‐based TBI and further enhance its safety and effectiveness.

## 5 CONCLUSION

This study confirms the clinical value of helical tomotherapy for TBI, providing reproducible and conformal dose delivery across diverse anatomical regions with minimal reliance on traditional field‐matching techniques. Through rigorous simulation‐to‐treatment QA, gamma analysis, and in vivo OSLD verification, we established a protocol for TBI that enhances patient safety, planning efficiency, and dosimetric precision. These findings support the broader implementation of tomotherapy‐based TBI in centers aimed at improving the therapeutic ratio in patients undergoing hematopoietic stem cell transplantation.

## CONFLICT OF INTEREST STATEMENT

The authors declare no conflict of interest.

## FUNDING INFORMATION

This study did not receive any grants from funding agencies in the public, commercial, or non‐profit sectors.

## ETHICAL APPROVAL

This research did not involve any direct patient involvement or modification of standard treatment protocols. All radiotherapy plans were generated as part of routine clinical workflows, and no prospective recruitment of human participants was undertaken. The study exclusively utilized de‐identified retrospective datasets and non‐interventional phantom‐based validation methods. According to institutional policies and prevailing national regulations, retrospective analyses involving anonymized data are exempt from the requirement of formal ethical committee approval.

## Supporting information



Supporting Information

## Data Availability

The data that support the findings of this study are available from the corresponding author upon reasonable request.
